# Potential Applications and Antifungal Activities of Engineered Nanomaterials against Gray Mold Disease Agent *Botrytis cinerea* on Rose Petals

**DOI:** 10.3389/fpls.2017.01332

**Published:** 2017-08-02

**Authors:** Yi Hao, Xiaoqian Cao, Chuanxin Ma, Zetian Zhang, Na Zhao, Arbab Ali, Tianqi Hou, Zhiqian Xiang, Jian Zhuang, Sijie Wu, Baoshan Xing, Zhao Zhang, Yukui Rui

**Affiliations:** ^1^Beijing Key Laboratory of Farmland Soil Pollution Prevention and Remediation, College of Resources and Environmental Sciences, China Agricultural University Beijing, China; ^2^Beijing Key Laboratory of Development and Quality Control of Ornamental Crops, Department of Ornamental Horticulture, China Agricultural University Beijing, China; ^3^Stockbridge School of Agriculture, University of Massachusetts, Amherst MA, United States; ^4^Department of Analytical Chemistry, The Connecticut Agricultural Experiment Station, New Haven CT, United States

**Keywords:** carbon nanomaterials, metal-based nanoparticles, roses, *Botrytis cinerea*, antifungal activities

## Abstract

Nanoparticles (NPs) have great potential for use in the fields of biomedicine, building materials, and environmental protection because of their antibacterial properties. However, there are few reports regarding the antifungal activities of NPs on plants. In this study, we evaluated the antifungal roles of NPs against *Botrytis cinerea*, which is a notorious worldwide fungal pathogen. Three common carbon nanomaterials, multi-walled carbon nanotubes, fullerene, and reduced graphene oxide, and three commercial metal oxidant NPs, copper oxide (CuO) NPs, ferric oxide (Fe_2_O_3_) NPs, and titanium oxides (TiO_2_) NPs, were independently added to water-agar plates at 50 and 200-mg/L concentrations. Detached rose petals were inoculated with spores of *B. cinerea* and co-cultured with each of the six nanomaterials. The sizes of the lesions on infected rose petals were measured at 72 h after inoculation, and the growth of fungi on the rose petals was observed by scanning electron microscopy. The six NPs inhibited the growth of *B. cinerea*, but different concentrations had different effects: 50 mg/L of fullerene and CuO NPs showed the strongest antifungal properties among the treatments, while 200 mg/L of CuO and Fe_2_O_3_ showed no significant antifungal activities. Thus, NPs may have antifungal activities that prevent *B. cinerea* infections in plants, and they could be used as antifungal agents during the growth and post-harvesting of roses and other flowers.

## Introduction

Roses are the most popular garden plant and cut flower, and they have been cultivated for several millennia (Debener and Linde, 2009). However, gray mold caused by the air-borne pathogen *Botrytis cinerea* is a devastating disease of rose worldwide, resulting in at least a 30% loss of production every year. In addition to roses, *B. cinerea* also infects various plant species, causing significant pre- and post-harvest damage to fruits, vegetables, and ornamentals (Ferrada et al., 2016; Jurick et al., 2017). And the process of *B. cinerea* infection could induce some pathogenic factors such as cell wall-degrading enzymes, reactive oxygen species (ROS), phytotoxin, oxalic acid, etc. (van Kan, 2006; Choquer et al., 2007; Williamson et al., 2007). It was reported that *B. cinerea* has become the second major plant pathogenic fungus after the rice blast fungus. Because of the serious damage caused by *B. cinerea* to plants and agricultural production, it was urgent to find fungicides, which could efficiently suppress the growth of *B. cinerea* (Kretschmer et al., 2009; Fernandez-Ortuno et al., 2016; Fan et al., 2017). Like *B. cinerea* there are many fungal pathogens and diseases severely impact crop yields, reducing the quality of agro-product, which could result in a worldwide threat to agricultural security (Fisher et al., 2012; Girard et al., 2016). Thus, the production and use of pesticides against fungal diseases has increased in recent years, which not only causes drug resistance but also produces new environmental risks (Gangemi et al., 2016). Consequently, it is urgent to investigate novel environmentally friendly fungicides that have efficient antifungal activities (Soylu et al., 2010; Fernandez-Acero et al., 2011).

Recently, the rapidly and continually developing field of nanotechnology has been widely applied to various disciplines, especially to environmental science. On the one hand, research has focused on assessing the environmental impact and ecological effects of nanoparticles (NPs), including their toxicological effects on plants, soil, and microorganisms (Hao et al., 2016; Mukherjee et al., 2016; Rui et al., 2016). On the other hand, some studies have focused on potential agricultural applications of low concentrations and low-toxicity nanomaterials. Servin et al. (2015) found that engineered nanomaterials can suppress plant disease, enhance crop yield, and play vital roles as fertilizers and pesticides. Similarly, Ditta and Arshad (2016) showed that nanomaterials can provide plants with more nutrients than conventional fertilizers. Additionally, nanomaterials can increase the storage time of vegetables and fruits, and improve animal immunity. Nanomaterials can also improve the water quality in fisheries (Peters et al., 2016). Thus, nanomaterials have huge potential to be used as novel nano-fertilizers, which can improve nutrient uptake and reduce agro-environmental pollution (Bindraban et al., 2015; Liu and Lal, 2015).

The potential applications of NPs as antifungal agents have also been investigated. For example, reduced graphene oxide (rGO) can inhibit the mycelial growth of the fungal pathogens *Aspergillus niger, Aspergillus oryzae*, and *Fusarium oxysporum* (Sawangphruk et al., 2012). Single-walled carbon nanotubes (SWCNTs), multi-walled carbon nanotubes (MWCNTs), fullerene (C_60_), and rGO have significant antifungal activities against *Fusarium graminearum* and *Fusarium poae* (Wang et al., 2014). SWCNTs can alter the oxidative enzyme activities of the saprotrophic white-rot fungi *Trametes versicolor* and *Phlebia tremellosa* (Berry et al., 2014). The toxicity of rGO toward the white-rot fungus *Phanerochaete chrysosporium* was dose-dependent. A low concentration of rGO promoted the growth of the fungus, while a high concentration induces a loss of fungal activity. Scanning electron microscope (SEM) observations showed that the fibers of the fungi were disrupted (Xie et al., 2016). For metal oxide NPs, Terzi et al. (2016) reported that nano-zinc oxide (ZnO) and nano-B_2_O_3_ suppressed the growth of molds, while nano-CuO (copper oxide) and nano-SnO_2_ prevented wood from undergoing fungal decay caused by *T. versicolor*. Silver (Ag) NPs can inhibit the colony formation of *Bipolaris sorokiniana* and *Magnaporthe grisea* in *in vitro* assays (Jo et al., 2009). De Filpo et al. (2013) reported that the photo-catalytic activities of titanium oxides (TiO_2_) NPs protected wood from *Hypocrea lixii* and *Mucor circinelloides* colonization, suggesting a potential antifungla mechanism. Hollow TiO_2_ NPs had the strongest antifungal activities against two potent phytopathogens, *Fusarium solani* and *Venturia inaequalis*, compared with pure and silver-doped TiO_2_ NPs. The NP concentrations and visible light intensity levels contribute to the antifungal effects (Boxi et al., 2016). Zn and ZnO showed antifungal activities against fungi (*Penicillium* and *Mucor* spp.; Swain et al., 2014). Cu NPs inhibited the growth of four major plant pathogenic fungi, *F. oxysporum, Curvularia lunata, Alternaria alternata*, and *Phoma destructiva*, mainly as a result of their large surface area to volume ratio, and can be used as neo-antifungal agents, protecting crops from plant pathogenic fungi (Kanhed et al., 2014).

Notably, previous reports of the antifungal activities of nanomaterial were based on *in vitro* assays, in which fungi were grown on artificial media, such as potato dextrose agar. Currently, the *in planta* effects of nanomaterials on the growth of fungal pathogens has not been addressed. The activities of fungicides vary between *in vitro* and *in vivo* assays, as illustrated by the antifungal activities of essential oils against *Sclerotinia sclerotiorum* and *Colletotrichum gloeosporioides* (Soylu et al., 2007; Hong et al., 2015).

In this study, the antifungal activities of six nanomaterials [ferric oxide (Fe_2_O_3_) NPs, CuO NPs, TiO_2_ NPs, MWCNTs, C_60_, and rGO] against *B. cinerea* were evaluated *in vitro* and *in vivo* conditions. The distinctive antifungal effects of the six nanomaterials on the morphology, size, and location of fungal hyphae on the rose petals were characterized by using SEM.

## Materials and Methods

### Sample Preparation and Characterization of Six Nanomaterials

Fe_2_O_3_, CuO, and TiO_2_ NPs were purchased as dry powders from Pantian Powder Material Company (Shanghai, China). MWCNTs were provided by the laboratory of Professor Wei Fei (Tsinghua University, China). C_60_ was ordered from Puyang Yongxin Fullerene Technology Co. Ltd. (Puyang, China), and rGO was purchased from Chengdu Organic Chemicals Co. Ltd. (Chengdu, China). All six nanomaterials were purified before being used in the experiments, three metal-based NPs, rGO and C_60_ were purified by material company before purchase, while MWCNTs were purified following the method of nitric acid purification (Andrade et al., 2013). A transmission electron microscope (TEM; JEM-2100, JEOL, Japan) was used for nanomaterial morphological and size determinations before the experiments. To prepare the TEM samples, the nanomaterials were dissolved and sonicated in ethanol and then dropped onto Cu grids.

All six nanomaterials were suspended in deionized water at concentrations of 50 and 200 mg/L for the stock suspensions, followed by sonication for 30 min to produce the required concentrations for subsequent experiments.

### Fungal Strain and Growth Conditions

The *B. cinerea* standard strain B05.10 used in this study was kindly provided by Dr. Jan van Kan (Wageningen University). It was routinely grown on potato dextrose agar at 22°C in the laboratory. *B. cinerea* conidia were harvested from 7- to 14-day-old fungal plates in 20 mL of water. The suspended conidia were washed with tap water. The resuspended conidia were then adjusted to a final concentration of 10^5^ conidia/mL in potato dextrose broth medium, with different nanomaterials.

### Antifungal Effect of Different Concentrations of NPs on Mycelial Growth *In Vitro* Conditions

The six nanomaterials, MWCNTs, rGO, C_60_, CuO NPs, Fe_2_O_3_ NPs, and TiO2 NPs, were suspended in 4‰ potato dextrose agar medium to the final concentrations of 5, 50, 100, and 200 mg/L, poured into Petri dishes of 60 mm in diameter. After cooling and solidification, well-cultivated 4-mm diameter *B. cinerea* colony was then inoculated in the center of medium, all the colonies were transferred to a climate-controlled room at 22°C. The diameter of mycelia was measured after 72-h incubation.

### Antifungal Effect of Different Concentrations of NPs on Mycelial Growth *In Vivo* Conditions

The six nanomaterials, MWCNTs, rGO, C_60_, CuO NPs, Fe_2_O_3_ NPs, and TiO_2_ NPs, were suspended in 4‰ water-agar to the final concentrations of 50 and 200 mg/L. For inoculation, cut rose (*Rosa hybrida* “Samantha”) flowers were harvested from a local commercial greenhouse and transported to the laboratory. One centimeter diameter discs were excised from the center of the petals using a hole punch. The petal discs were gently rinsed by deionized water, placed on 4‰ water-agar plates amended with or without nanomaterials. Flower petals were carefully placed in potato dextrose agar media containing different concentrations of NPs, and then the suspension of conidia (2 μL, 10^5^ conidia/mL in potato dextrose broth medium) independently containing different nanomaterials was dropped on the petals so as to determine the effect of NPs on fungi infection. In this way, we can mimic the microenvironment of rose petal, namely the flower petals were soaked by NP suspensions in both sides. Additionally, the suspension of conidia and NPs dropped in petals could make sure the even distribution of NPs around conidia. Deionized water and conventional fungicide procymidone containing same concentration of conidia was used in petal as negative control and positive control, respectively, in the second and the third group. After inoculation, Petri dishes were immediately transferred to a climate-controlled room at 22°C. After 72 h, the infection results were photographic record and the fungal plaque diameter was measured.

### Characterization of Antifungal Effect of NPs on Hyphal Morphology

The inoculated petals were observed 72 h after inoculation. A SEM (Phenom ProX, Phenom, Netherlands) was used to determine the morphology, size, and location of *B. cinerea* on the rose petals. The *B. cinerea* infections on the rose petals were photographically recorded (α7RII, Sony, Japan).

### Fungi Infection in the Whole Cut Flowers

Rose flowers were harvested at a local commercial greenhouse, and the branches of cut flowers were soaked in deionized water. Twelve droplets of a suspension of conidia (2 μL, 10^5^ conidia/mL in potato dextrose broth medium) independently containing the six nanomaterials were dropped in the outmost two petals, the treated flowers were then placed in a climate-controlled room, in which the temperature was set at 22°C and the moisture was controlled at 90%. After 96 h the infection, flower images were photographic recorded and the total fungal plaque areas was measured.

### Statistical Analyses

The experiments were conducted in triplicate. Statistical analysis, a one-way analysis of variance followed by Duncan’s multiple range test (*P* ≤ 0.05), was conducted using SPSS 19.0 for windows (SPSS, Chicago, IL, United States).

## Results

### Characterization of Six NPs

**FIGURE 1:**
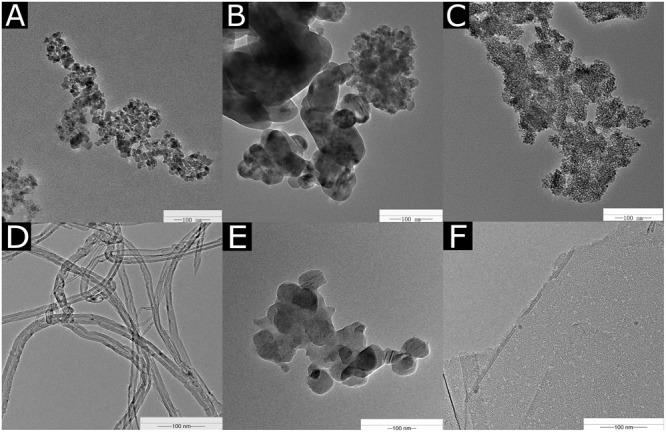
TEM images of Fe2O3 NPs **(A)**, CuO NPs **(B)**, TiO2 NPs **(C)**, MWCNTs **(D)**, C60 **(E)**, and rGO **(F)**.

The TEM images of the NPs are shown in **Figure 1**. The diameters of the Fe_2_O_3_ NPs ranged from 40 to 100 nm in the TEM images (**Figure 1A**). The diameter of the CuO NPs used in our experiment was 20–30 nm (**Figure 1B**). The average size of the TiO_2_ NPs was about 20 nm (**Figure 1C**). The MWCNTs were agglomerated and mingled (**Figure 1D**) with a cross-sectional diameter of 20–30 nm. C_60_ was spherical in shape and tended to aggregate (**Figure 1E**), and the C_60_ diameter was approximately 50 nm. The diameter of rGO was about 500 nm, and the thickness of a single layer ranged from 0.55 to 3.74 nm (**Figure 1F**).

### Antifungal Effect of Different Concentrations of NPs on Mycelial Growth *In Vitro* Conditions

After 72-h growth of *B. cinerea* on potato dextrose agar medium mixed with six nanomaterials at the concentrations of 5, 50, 100, and 200 mg/L, the diameter of mycelia was measured and illustrated in **Table 1**. The results showed that all the 5 mg/L NP treatment had no obvious inhibited effects on mycelia growth. Fe_2_O_3_, CuO NPs, and MWCNTs significantly suppressed mycelia growth at the concentration of 50, 100, and 200 mg/L, while TiO_2_ had no influence on mycelia growth regardless of the exposure doses. The inhibited effect of rGO was significant in the concentration of 100 and 200 mg/L. Interestingly, compared with other treatments, the diameter of mycelia significantly decreased only in the treatment of 50 mg/L. These results indicated that NPs had antifungal effects on potato dextrose agar, considering the inhibited effects on mycelia growth, the concentrations of 50 and 200 mg/L was selected and used in our next fungal infection experiments on rose.

**Table 1:** The effects of different concentrations of NPs on the mycelial growth of *B. cinerea.*

Treatment	Concentration (mg/L)	Diameter (cm)
Control	0	3.9 ± 0.05a
Fe_2_O_3_	5	3.66 ± 0.06ab
	50	3.42 ± 0.28b
	100	3.38 ± 0.03b
	200	3.58 ± 0.19b
CuO	5	4.08 ± 0.10a
	50	3.20 ± 0.15b
	100	3.23 ± 0.26b
	200	3.40 ± 0.09b
TiO_2_	5	3.77 ± 0.08a
	50	3.80 ± 0.05a
	100	3.78 ± 0.03a
	200	3.81 ± 0.13a
C_60_	5	4.05 ± 0.13a
	50	3.13 ± 0.23b
	100	3.92 ± 0.34a
	200	3.74 ± 0.09a
MWCNTs	5	3.87 ± 0.16a
	50	3.63 ± 0.10b
	100	3.46 ± 0.07b
	200	3.47 ± 0.08b
rGO	5	4.05 ± 0.1a
	50	3.97 ± 0.3a
	100	3.4 ± 0.23b
	200	3.65 ± 0.1b

*Mean values, for each NP, with the same letters represent values that are not significantly different according to Duncan’s multiple range test (*P* ≤ 0.05).*

### Effects of Fungal Infection on Rose Petals and the Whole Cut Flowers

**FIGURE 2:**
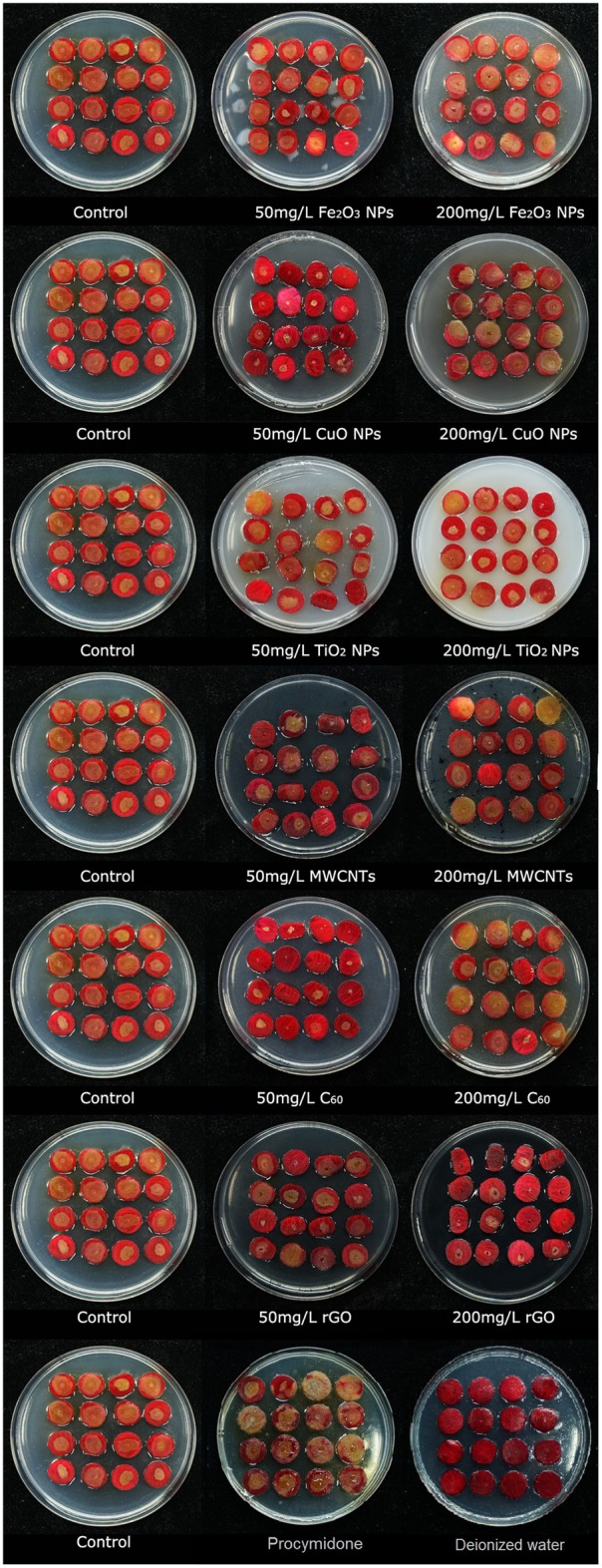
Photographs of rose petals after being infected by *B. cinerea* co-cultivated with different nanoparticles, Fe2O3 NPs, CuO NPs, TiO2 NPs, MWCNT, C60, and rGO, at concentrations of 50 and 200 mg/L in the PDA mixed with same type of NPs.

After a standard 3-d infection test with *B. cinerea*, the infection and resistance levels of rose to *B. cinerea* under all NP treatments were observed and evaluated (**Figure 2**). The color of the rose petal changed when subjected to CuO NPs, MWCNTs, and rGO treatments from red to dark red compared with the control. Based on the images in **Figure 2**, the most dramatic changes were those of the *B. cinerea* colony diameters after being treated 50 mg/L of Fe_2_O_3_, CuO, and C_60_, and 200 mg/L of MWCNTs and rGO, which significantly inhibited the *B. cinerea* infection.

**FIGURE 3:**
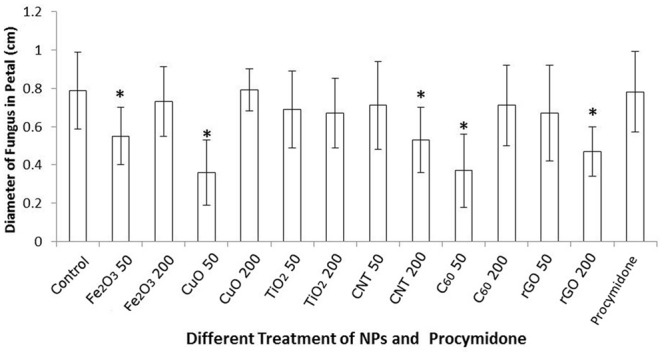
Colony diameters (cm) of *B. cinerea* co-cultured with six different NPs, Fe_2_O_3_ NPs, CuO NPs, TiO_2_ NPs, MWCNT, C_60_, and rGO, at concentrations of 50 and 200 mg/L 72 h after infection. Significant differences (*P* < 0.05) between the NP treatments and the control are marked with “asterisks.”

The colony diameters of *B. cinerea* on petals are presented in **Figure 3**. The results are consistent with the observations and photographs. C_60_, CuO NPs, and Fe_2_O_3_ NPs significantly reduced the fungal colony diameters at the 50 mg/L concentration, MWCNTs and rGO also suppressed the growth of *B. cinerea* at the 200 mg/L concentration. Because of the inhibitory effects on *B. cinerea* found in this study, and their environmentally friendly nature (Lee et al., 2016; Yang et al., 2016), carbon nanomaterials (MWCNTs, C_60_, and rGO) are the most ideal materials for promoting plant fungal disease resistance. Compared with different NP treatments, the inhibited effects of conventional fungicide procymidone was not obvious, there was no significant difference of colony diameter between the treatment of procymidone and control. Meanwhile, as the negative control, there was no obvious change of petal in the treatment with deionized water.

However, considering the existence of different NPs both in the PDA media and conidia suspension, this promoting antifungal effect might also result from the interaction between rose and NPs instead of the inhibited effect of NPs on *B. cinerea*, so we conducted an experiment to exclude this possibility. Rose petals were placed in the potato dextrose agar media without any NP additions and then the droplets of the conidia suspension was added (the same as the former experiment), after 72-h incubation the infection result was consistent with the treatment of NP exposures both in the PDA media and conidia suspension (Supplementary Figures S1, S2). This result indicated that the inhibited effect was mainly because of the interaction between fungi and NPs.

**FIGURE 4:**
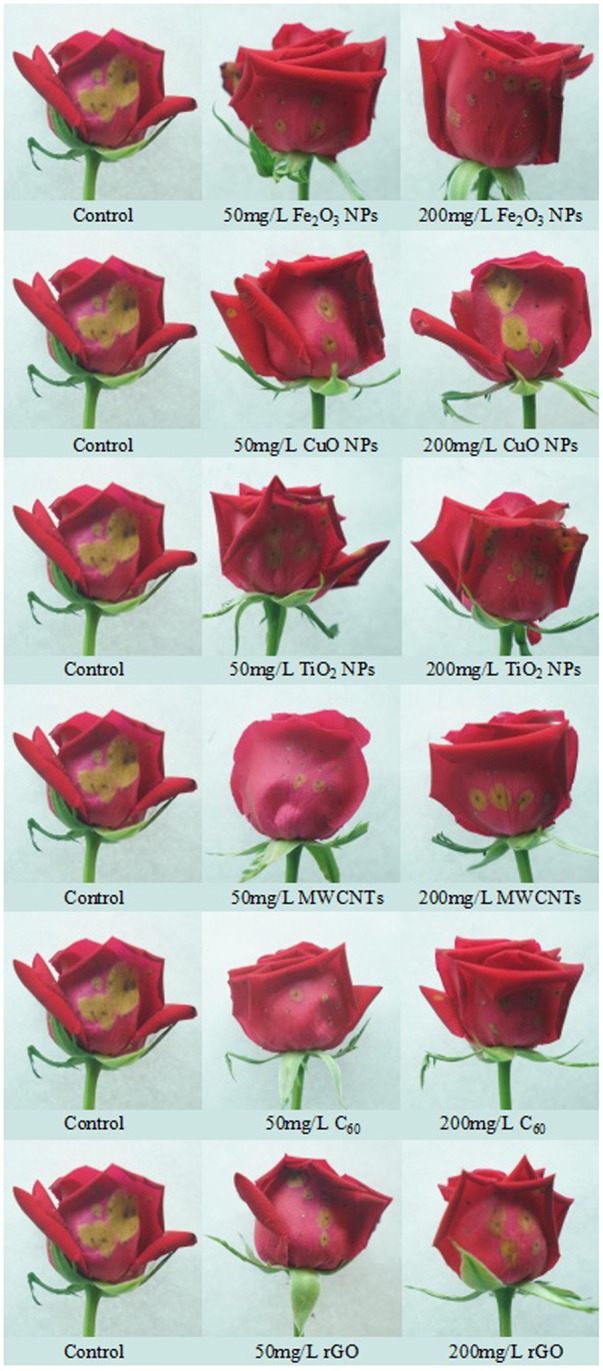
Photographs of the whole roses after being infected by *B. cinerea* co-cultivated with different nanoparticles at concentrations of 50 and 200 mg/L.

Under natural conditions, the infection of *B. cinerea* generally happens after rose harvests, especially in the processes of transportation and preservation. To evaluate the antifungal effect in the natural environment, we then measured the colony area of *B. cinerea* in the whole cut flowers under moisture conditions. The results were consistent with the ones from the petal experiment (**Table 2** and **Figure 4**). Notably, MWCNTs showed distinguished antifungal effect both in the concentrations of 50 and 200 mg/L compared with the detached petal experiment, implying that the inhibited effects of carbon-based nanomaterials might be improved in the natural conditions.

**Table 2:** Colony area of *B. cinerea* co-cultured with different NPs at concentrations of 50 and 200 mg/L in the whole cut flowers (cm^2^).

Treatments	Concentration (mg/L)	Area (cm^2^)
Control	0	5.56 ± 0.97a
Fe_2_O_3_	50	3.33 ± 2.38a
	200	4.21 ± 2.39a
CuO	50	3.02 ± 0.98b
	200	3.54 ± 1.16ab
TiO_2_	50	5.091 ± 1.47a
	200	5.83 ± 0.42a
C_60_	50	3.60 ± 0.53b
	200	5.65 ± 1.26a
MWCNTs	50	2.08 ± 1.34b
	200	1.63 ± 0.97b
rGO	50	4.07 ± 0.36b
	200	4.04 ± 0.54b

*Mean values, for each NP, with the same letters represent values that are not significantly different according to Duncan’s multiple range test (*P* ≤ 0.05).*

### SEM Observations of Rose Petals after Fungal Infection

**FIGURE 5:**
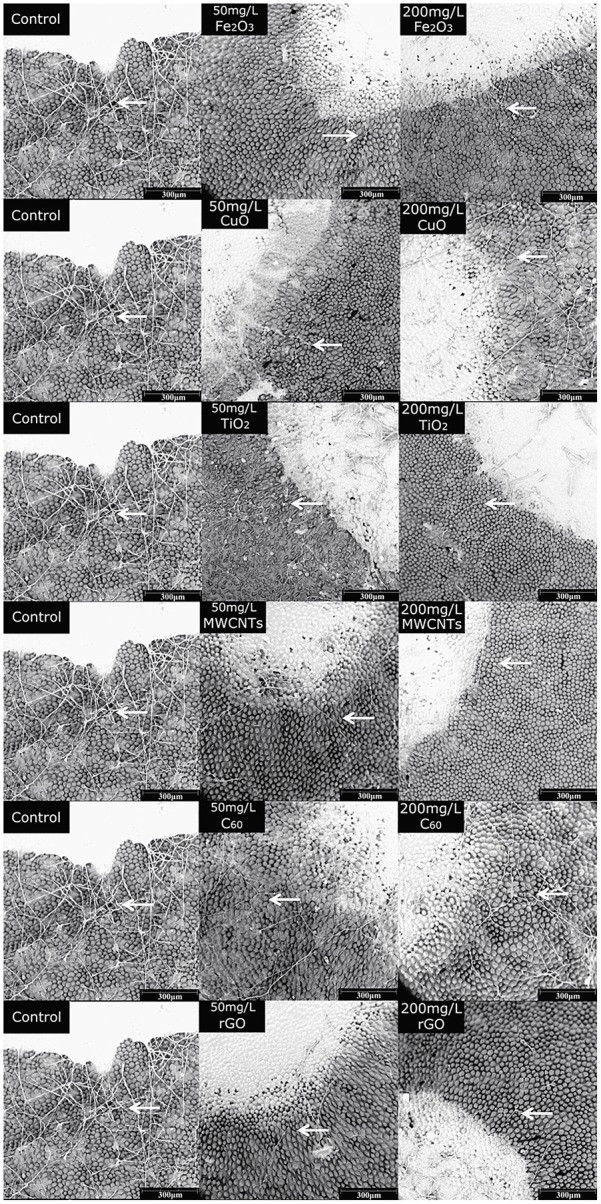
SEM images of rose after petals infected with *B. cinerea* co-cultivated with different nanoparticles, Fe_2_O_3_ NPs, CuO NPs, TiO_2_ NPs, MWCNT, C_60_, and rGO, at concentrations of 50 and 200 mg/L. Compared with the control, the fungi exposed to NPs were scarce, the hyphae (arrows) were slender and fragile.

The rose petals treated with different NPs were observed by SEM at 72 h post inoculation. As shown in **Figure 5**, the *B. cinerea* at the edge of the lesions were phenotypically different. Compared with the control, the fungi exposed to NPs were scarce and lacked viability. The hyphae were slender and fragile, indicating the inhibitory effects of NPs on *B. cinerea*’s growth and suggesting that they have antifungal properties.

## Discussion

*In vitro* and *in planta* assays are two major methods for evaluating the effects of potential antifungal agents, such as metabolites (Lecomte et al., 2012; Zhang et al., 2013), commercial fungicides (Dal Maso et al., 2014), biological control agents (Pane and Zaccardelli, 2015; Yao et al., 2016), and plant essential oils (Hong et al., 2015; Varo et al., 2017). Compared with *in vitro* assays, *in planta* experiments have more practical significance and are widely used in screening efficient fungicides and assessing antifungal activities (Dal Maso et al., 2014; Hrabětová et al., 2017; Varo et al., 2017). In our study, the inhibitory effects of NPs on fungi were determined on detached rose petals after 72 h.

In our study, three carbon nanomaterials inhibited the *in planta* growth of *B. cinerea*. The inhibitory effects of rGO on *B. cinerea* were obvious at both low and high concentrations, while only 200 mg/L of rGO yielding significant antifungal results. Graphene sheets may disrupt fungal structures and damage the cell wall with their sharp edges (Xie et al., 2016), producing oxidative stress (Akhavan and Ghaderi, 2012), showing further antifungal effects. These toxicological mechanisms could explain the suppressive effects of rGO on *B. cinerea*. After exposure to MWCNTs, the effects of fungi on rose petals were also inhibited, especially after treatment with 200 mg/L MWCNTs, while the suppressive effects of 50 mg/L MWCNTs was not significant, which was different from the antifungal effects of 50 mg/L rGO. C_60_ significantly inhibited the growth of *B. cinerea* only at the 50 mg/L concentration. Similar to our results, Wang et al. (2014) found that MWCNTs and rGO could inhibit the growth of *F. graminearum* by suppressing spore germination at concentrations of 62.5, 125, 250, and 500 mg/L, while the inhibitory effect of C_60_ was not obvious at the same concentrations. Carbon nanomaterials can tightly contact spores, forming CNM-spore aggregates in the co-culture system through van der Waals forces (Wang et al., 2014). These aggregates effectively suppress the activities of spores by influencing the growth of fungi and, as a result, may inhibit *B. cinerea* infections on roses. The aggregative effects between carbon nanomaterials and spores also indicated the crucial roles of the physical properties of nanomaterials, such as van der Waals force.

Similar to carbon nanomaterials, Fe_2_O_3_ and CuO NPs also showed in planta antifungal effects on rose petals, which implied potential applications to protect flowers from fungal infection. Interestingly, the inhibitory effects of CuO and Fe_2_O_3_ NPs were significant only at the 50 mg/L concentration, but were not obvious at 200 mg/L. The antifungal effects of CuO NPs mainly resulted from the release of Cu^2+^, which is a major toxicity mechanism of Cu-based nanomaterials, such as Cu, CuO, and Cu_2_O NPs (Kanhed et al., 2014; Zarzuela et al., 2017). As in our results, CuO NPs also inhibited the decay of wood by *Gloeophyllum trabeum* and *T. versicolor*, which indicated the potential application of CuO NPs as fungicides (Terzi et al., 2016). Similarly, Fe_2_O_3_ NPs suppressed the infection of *B. cinerea* significantly at the 50 mg/L concentration, while the antifungal effects were not obvious after exposure to 200 mg/L Fe_2_O_3_ NPs. TiO_2_ NPs can induce cell damage in *Pichia pastoris* by impairing the ROS-associated scavenging system, especially the cycle and regulation of glutathione, which resulted in the accumulation of ROS (Liu et al., 2016). However, in our study, the antifungal effects of TiO_2_ NPs were not obvious at either 50 or 200 mg/L concentrations. Considering the similar size of TiO_2_ NPs, these different results may reflect the different sensitivities of *P. pastoris* and *B. cinerea*. The various sensitivities of different fungi to metal NPs emphasize the need for screening antifungal NPs *in planta*. Besides the efficient antifungal effects of NPs investigated in our study, other NPs also showed antifungal property against *B. cinerea*. For example, Ag NPs could significantly inhibit the growth of *B. cinerea* at concentration of 25 mg/L, and the inhibition effect was dose-dependent (Derbalah et al., 2012). Additionally, Ag NPs could also suppress other fungi such as *A. alternata, S. sclerotiorum, Macrophomina phaseolina, Rhizoctonia solani*, and *C. lunata* (Kovačec et al., 2017). ZnO NPs was another antifungal metal NP, it was reported that ZnO NPs could significantly decrease the growing activity of *B. cinerea* at concentrations greater than 3 mmol/L via affecting cellular functions especially deforming the fungal hyphae (He et al., 2011). Thus, nanomaterials could become an idealistic substitute for the conventional fungicide.

SEM observation after 72-h incubation further indicated the inhibition effects of NPs on fungi growth. It was obvious that after NP treatment, the number of mycelia was decreased comparing with control, meanwhile the growth state of mycelia was not active in the treatment of NPs, as the diameter of mycelia was considerably decreased compared with control (**Figure 5**). The decrease of mycelia number could be ascribed to the inhibition effects of the carbon nanomaterials such as MWCNTs, SWCNTs, GO, and rGO which could suppress spore germination by blocking the water channels of spores (Wang et al., 2014). Meanwhile metal dissolution should be another possible factor that participated in inhibiting fungi growth in rose petal (Zarzuela et al., 2017).

To date, several mechanisms have been proposed to illustrate the inhibited effect of NPs on various fungi. On the one hand, the closely adherence of NPs to the cell surface could induced a serious changes, such as physical cutting, water channel blocking, membrane permeability increasing, and even the penetration of NPs into fungus cells, which could result in cell death (Wang et al., 2014). On the other hand, the existence of NPs could induce ROS accumulation via impairing the ROS-scavenging system of fungi (Liu et al., 2016). Furthermore, the ions release of metal-based NPs was another mechanism of NP toxicity to fungi (Zarzuela et al., 2017).

In summary, the results of our study indicated the efficient antifungal activities of carbon nanomaterials and metal NPs on *B. cinerea*, pointed out the potential for the use of nanomaterial against pathogenic fungi on flowers.

## Conclusion

Taken together, as potential fungistats, C_60_ and CuO NPs could effectively suppress *B. cinerea* infections and improved flower quality at relatively low concentrations (50 mg/L), which could reduce the environmental risks resulting from NP release and reduce the production costs of novel NP fungistats. Our study showed the huge potential of NP applications in the field of horticulture because of their antifungal properties. In our study, we tried to increase the NP efficiency on inhibiting fungi growth at a relative low exposure dose to minimize the NP residue, meanwhile previous studies also pointed out NPs such as MWCNTs and Fe_2_O_3_ NPs have relatively lower toxicity, and could stimulate the growth and development of plants (Khodakovskaya et al., 2013; Rui et al., 2016). Considering the complex toxicity mechanisms of different NPs, any NP applications should be carefully evaluated for the potential environmental risks.

## Author Contributions

YR and ZZ proposed the present study. YH, ZZ, CM, BX, and YR designed the experiments. YH, XC, ZeZ, AA, NZ, JZ, SW, ZX, and TH performed the experiments and analyzed the data. YH, ZZ, and YR wrote the paper. All authors have read and approved the final manuscript.

## Conflict of Interest Statement

The authors declare that the research was conducted in the absence of any commercial or financial relationships that could be construed as a potential conflict of interest.
