# Supplementary material for: Potential Applications and Antifungal Activities of Engineered Nanomaterials against Gray Mold Disease Agent Botrytis cinerea on Rose Petals
Source: Front Plant Sci. 2017 Aug 2;8:1332. doi: 10.3389/fpls.2017.01332 (PMC5539092; doi:10.3389/fpls.2017.01332)
Supplement: Supplementary file 1 [file Image_1.pdf]

Potential applications and antifungal activities of engineered nanomaterials against gray mold disease agent *Botrytis cinerea* on rose petals

Yi Hao[1,†], Xiaoqian Cao[2,†], Chuanxin Ma[3,4], Zetian Zhang[1], Na Zhao[2], Arbab Ali[1], Tianqi Hou[1], Zhiqian Xiang[1], Jian Zhuang[1], Sijie Wu[1], Baoshan Xing[3], Zhao Zhang[2]*, and Yukui Rui[1,3]*

[1]Beijing Key Laboratory of Farmland Soil Pollution Prevention and Remediation, College of Resources and Environmental Sciences, China Agricultural University, Beijing 100193, China

[2] Beijing Key Laboratory of Development and Quality Control of Ornamental Crops, Department of Ornamental Horticulture, China Agricultural University, Beijing 100193, China

[3]Stockbridge School of Agriculture, University of Massachusetts, Amherst, MA 01003, USA

[4]Department of Analytical Chemistry, The Connecticut Agricultural Experiment Station, New Haven, CT 06504, USA

*Corresponding author:

Yukui Rui: ruiyukui@163.com; Tel: 86-10-62733470; Fax: 86-10-62733470

[†] These authors contributed equally to this work.

Number of Pages: 3
Number of Figures: 2

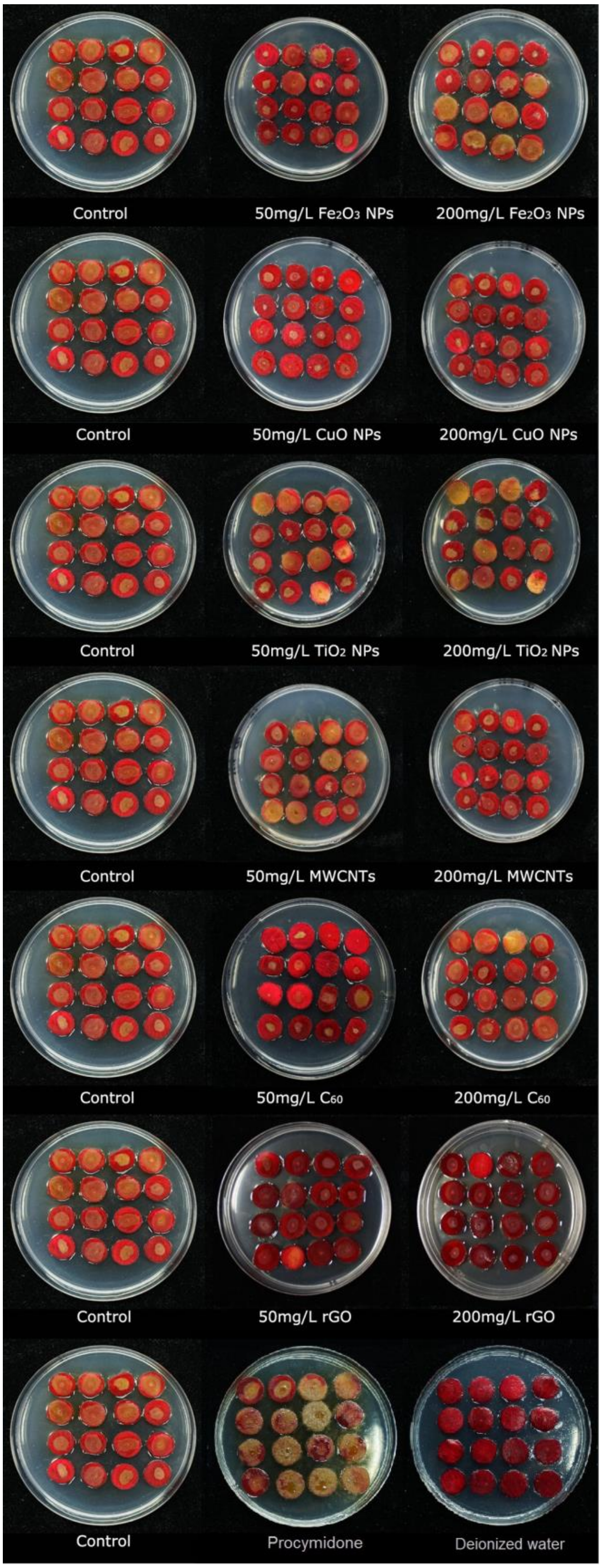

Figure S1. Photographs of rose petals after being infected by *B. cinerea* co-cultivated with different nanoparticles at concentrations of 50 and 200 mg/L placed in the pure PDA.

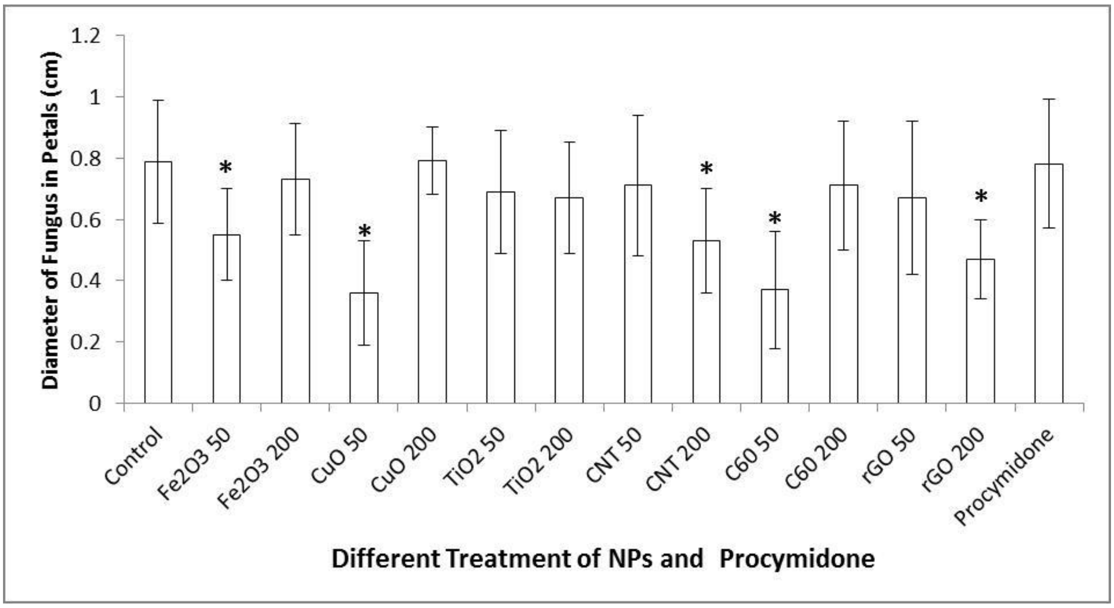

Figure S2. Colony diameters (cm) of *B. cinerea* co-cultivated with different nanoparticles at concentrations of 50 and 200 mg/L placed in the pure PDA. Significant differences ($p < 0.05$) between the NP treatments and the control are marked with "asterisks".
